# Spectroscopic techniques for authentication of animal origin foods

**DOI:** 10.3389/fnut.2022.979205

**Published:** 2022-09-20

**Authors:** Vandana Chaudhary, Priyanka Kajla, Aastha Dewan, R. Pandiselvam, Claudia Terezia Socol, Cristina Maria Maerescu

**Affiliations:** ^1^College of Dairy Science and Technology, Lala Lajpat Rai University of Veterinary and Animal Sciences, Hisar, India; ^2^Department of Food Technology, Guru Jambheshwar University of Science and Technology, Hisar, India; ^3^Division of Physiology, Biochemistry and Post-Harvest Technology, ICAR–Central Plantation Crops Research Institute, Kasaragod, India; ^4^Department of Genetics, University of Oradea, Oradea, Romania

**Keywords:** dairy products, authentication, animal based products, spectroscopic techniques, pre-processing, chemometrics

## Abstract

Milk and milk products, meat, fish and poultry as well as other animal derived foods occupy a pronounced position in human nutrition. Unfortunately, fraud in the food industry is common, resulting in negative economic consequences for customers as well as significant threats to human health and the external environment. As a result, it is critical to develop analytical tools that can quickly detect fraud and validate the authenticity of such products. Authentication of a food product is the process of ensuring that the product matches the assertions on the label and complies with rules. Conventionally, various comprehensive and targeted approaches like molecular, chemical, protein based, and chromatographic techniques are being utilized for identifying the species, origin, peculiar ingredients and the kind of processing method used to produce the particular product. Despite being very accurate and unimpeachable, these techniques ruin the structure of food, are labor intensive, complicated, and can be employed on laboratory scale. Hence the need of hour is to identify alternative, modern instrumentation techniques which can help in overcoming the majority of the limitations offered by traditional methods. Spectroscopy is a quick, low cost, rapid, non-destructive, and emerging approach for verifying authenticity of animal origin foods. In this review authors will envisage the latest spectroscopic techniques being used for detection of fraud or adulteration in meat, fish, poultry, egg, and dairy products. Latest literature pertaining to emerging techniques including their advantages and limitations in comparison to different other commonly used analytical tools will be comprehensively reviewed. Challenges and future prospects of evolving advanced spectroscopic techniques will also be descanted.

## Introduction

Food adulteration, which means the incorporation of inferior quality ingredients/components or the elimination of important dietary constituents, is certainly a traditional practice of producing food. When it comes to choosing food commodities, consumers require accurate and unbiased information. Consumer preferences are typically influenced by lifestyle, and socio-economic factors like vegetarians prefer eating fresh organic produce, while some non -vegetarians abstain from eating pork and certain portions of muscle foods, and obviously, the health concerns as the person suffering from different types of food intolerances and allergies will refrain from concerning food products. Consumer knowledge has grown to the point that commercially oriented adulteration is now recognized as a severe public health threat (1). Adulteration in food commodities can emanate by substituting an ingredient/constituent with cheap and inferior quality ingredients incorporating illicit substances, arbitrarily prolonging shelf stability, stating misleading processes, spreading misinformation about ingredients, and falsifying records about manufacturing origin (2). Food authenticity refers to the process of assessing food for quality, safety, and acquiescence with label information, customer protection legislation, and technical specifications (1). The growing availability of various food products, as well as incidents of adulteration resulting in significant pecuniary losses and negative impact on human well-being, has sparked widespread apprehension about food falsification’s huge detrimental impact on the worldwide chain of food supply. Market globalization has expanded food variety due to exchange among domestic and international markets from different regions around the globe to an extent where ascribability in the food production chain, as well as transportation, has been compromised. Consequently, food authentication is becoming a perilous apprehension in the arena of the food sector, as end-product value is affected by the actions of growers, business operators, manufacturers, customers, and all others engaged directly or indirectly involved in processing, storage, distribution and finally consumption. Food processors and business operators have to abide by labeling guidelines, which oblige them to disclose the main ingredients within every commodity. Customer’s value readily construed quality indicators like certified quality labels, territorial indications, or guarantee assurance seals, as well as necessary standard information like best before dates. Authentication techniques for food are designed to distinguish between fake and original commodities, eliminate prejudicial competition in the marketplace, and keep away consumers from fraud. To meet customer and food safety requirements, researchers are looking at creating and designing of effective techniques for detection of food adulteration and frauds. The efforts about the prevention of food are mostly centered on tracking the complete food supply chain right from raw material selection to processing, storage, and distribution. However, numerous traditional food risk, as well as fraud assessment methodologies, be unsuccessful in sleuthing or envisaging food authentication. Recently, varied spectroscopic techniques have emerged as a promising approach to resolving the issue of food authentication and fraud recognition. Near-infrared (NIR), mid-infrared (MIR), Fourier transform infrared (FTIR), nuclear magnetic resonance (NMR) or Raman spectroscopy, or hyperspectral imaging, are some of the spectroscopic techniques employed for food authentication so far (3) (Table 1).

**TABLE 1:** Various spectroscopic techniques applied for animal origin food authentication and adulteration with their strength and pitfalls.

Spectroscopic technique	Working principle	Based on the phenomenon	Outcomes	Application	Strength	Pitfalls	References
Terahertz Spectroscopy	Employs magnetic field depicting frequency ranging from hundred gigahertz to many terahertz	Vibrational transitions	Helps in depicting the qualitative and quantitative information pertaining to food constituents	- Detection of extraneous matter (stone, nail, plastic, hair etc.) in food products - To identify antibiotics, microorganisms, toxins, additives, and hazardous substances - For measuring the moisture content	- Non-destructive - Reagent free, fast and simple - Safe to use - No sample preparation required -Accurate - Longer THz spectral band	- Due to substantial suppression of THz signals in the presence of water, THz imaging is confined to dry food matrices. - High cost of THz source as well as detector - Non-uniform texture of food cause scattering in transmission and reflection mode - Constrained penetration of radiations in liquid as compared to solid - Requires competent chemometric approaches to reduce duplicated THz spectroscopic and imaging data	(77–79)
Laser-Induced Breakdown Spectroscopy	Food sample is exposed to intensified and highly concentrated laser pulse, which generates a tiny stream of plasma composed of excited atoms and ions. When these atoms/ions descend back to their ground state, they emit specific wavelengths of light, further collected by a spectrometer. The spectrum produced is examined for emission lines and the material can be identified and quantified.	Optical/Atomic emission	- helpful in characterization as well as identification of food materials	- To detect adulteration - To determine geographical origin	- Provides concurrent multi-elemental concentration of an analyte in all forms of matter - Minimal or no sample preparation	- Lower reproducibility rate of results - Not able to detect elements present in lower limits	(80, 81)
Hyperspectral imaging	Spectral image acquisition at few discrete and narrow wavebands in spatial direction	Absorption, transmission or scattering of electromagnetic radiations of specific wavelength characteristic of compounds and acquisition single or multiple images	Detect individual traits or features directly connected with quality	- Authenticate origin -Evaluate chemical and physical properties	-Single or multiple images - Non-destructive techniques - Can accurately analyze number of compounds in a single measurement - Can be implemented on a commercial level over a conveyer belt	- Expensive - Require high performance digital camera - Require high speed hardware - Can be complicated - Large size of dataset requires significant amounts of storage space	(82)
NMR	Phenomenon of absorption and emission of energy in the radiofrequency range of the electromagnetic spectrum	Numbers of resonating nuclei are measured as signals that are directly used for quantitative purpose.	Detect different classes of chemical compounds simultaneously	-Unveil erudite frauds - Address geographical source - Disclose possible for authentication markers	- Powerful tool for food -Characterization and authentication efficiently trace fraudulent labeling -No special requirement for sample preparation	- Not suitable for analysis of non-homogenous samples like milk - Presence of paramagnetic metals in foods like meat, spices limit its application resulting in poor resolution spectra	(83)
Raman spectroscopy	Optical measurement of energy transfer of light particle from the molecules present in the sample material	Spectrum is obtained by the molecular vibrations while bond extension and bending caused due to the variation in polarizability	Characterize molecular structure of chemical substances - Identify functional groups identification of chemical molecules - Distribution of chemical compounds, and their structure	- Adulteration detection in milk and dairy products, beverages, honey and grain - Detection of species fraud in meat and fish product	-Non-destructive technique - Highly specific -provides unique fingerprint of sample - Rapid and higher accuracy - Suitable for packed product (thin plastic or glass) - Suitable for aqueous solution -Suitable for large number of samples	-Sensitive - Requires high level of optimization for detection - Fluorescence of impurities or sample can interfere in Raman spectrum - Complex data analysis requires skilled technicians	(84)
Near Infrared spectroscopy	Measure the absorption of electromagnetic waves ranging between 780–2500 nm when subjected on sample	Variation in absorption at a particular wavelength depends on the composition of food, geographical origin, variety or genotype	- Peculiar spectrum of each food allows its identification and differentiation	-Freshness, shelf-life, authenticity, mislabeling of seafood - Chemical composition and microbiological evaluation of seafood - Quantitative and qualitative evaluation of meat and meat based product	-Low cost - Measure target rapidly - Non-destructive technique - Suitable for online analysis	- Low efficiency in certain food analysis	(85)
Vibrational spectroscopy	Measure the amount of incident light on the sample that can be absorbed, scattered, transmitted or reflected during interaction	Interaction of electromagnetic radiations and vibrational or excited states of atomic nuclei	-Identification/authentication qualitative analysis detection of food composition and characteristics	-Authentication of food commodities - Compositional studies	- Non-destructive technique -Rapid and simple - Suitable for online and in-line analysis	- Hardly selective - Difficult to acquire accurate and robust models - Reference methods are required for evaluation of specific parameters	(86)
UV-Vis spectroscopy	Measures the amount of light absorbed by the sample at the particular wavelength of UV-Vis range	Beer’s Law where concentration of solute is directly proportional to the amount of light absorbed	- Absorbed spectra provide fingerprints of compounds	-Authenticate the food compounds based on their native absorption spectrum - Geographic classification - Adulterant detection in various food categorical	- Applicable for wide range of compounds - Good sensitivity and accuracy - Moderate selectivity	-Low sensitivity and selectivity -Not a useful tool for qualitative analysis of organic compounds	(1)

Large volume of experimentation data is obtained from food experiments either in quantitative or in qualitative form. Maneuvering of such large quantity of data utilization of multivariate statistical tools has been increasingly embraced by food technologists. Chemometrics, is typically used when the dataset is substantial and complex in regard to number of samples, types as well as responses. The outcomes are applied for food authentication in regard to geographical origin, food fraud or to track adulteration in different food products. Additionally, chemometrics is helpful in bridging the gaps in transdisciplinary data required for sound scientific conclusions. A significant amount of information is generated by spectroscopic methods, which can be effectively utilized by applying multivariate mathematical and statistical (chemometric) methods (4). This review investigates the feasibility and potency of different non-destructive methods for animal based food commodities authentication that have the possibilities to contribute the dairy, meat, seafood and poultry processing industry with reliable and accurate real-time monitoring in short span. As a result, the purpose of this compiled literature is to provide an insight into current non-destructive spectroscopic technologies for animal based processed product authentication.

## Latest spectroscopic techniques used for authentication of animal origin foods

### Terahertz spectroscopy

**FIGURE 1:**
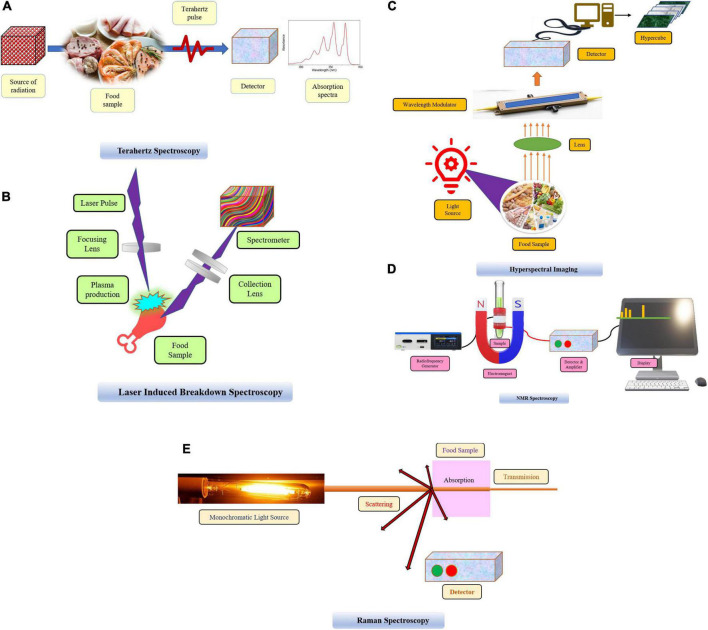
**(A)** Terahertz spectroscopy. **(B)** Laser induced breakdown spectroscopy. **(C)** Hyperspectral imaging. **(D)** NMR spectroscopy. **(E)** Raman spectroscopy.

Terahertz (THz) spectroscopy is gaining traction as a valuable method for food authentication in the field of food technology and processing. It assesses the properties of matter under an electromagnetic field. Terahertz refers to the region of electromagnetic radiation spanning in between microwave and infrared (IR) region (far IR). It is represented by a frequency lying between 0.1–10 THz, which corresponds to wavelengths from 3000 to 30 μm (5). THz may be accredited by different terminologies such as T-waves, terahertz gap, terahertz light, terahertz waves, T-lux., or T-light. THz wave explorations and utilization are still in their inception stage when compared to the comparatively well-developed and widely used conventional spectroscopic and imaging techniques. There are three types of THz spectroscopy, namely THz time-domain spectroscopy (THz-TDS), time-resolved THz spectroscopy (TRTS) and THz emission spectroscopy (TES) (6). A typical THz system comprises of a source, sample and a detector (Figure 1A). With the evolvements of THz sources and detectors in recent decades, this approach has shown significant promise as a novel and powerful non-destructive technique for real-time monitoring and quality control of food processes (7). The THz wave have the luxury of being able to be transmitted through substances opaque to visible light and having a photon energy of 1–100 meV, which is within the critical energy range for various materials and biomolecules. Numerous chemical compounds and microbial components have distinct spectrum patterns at THz frequencies, which can be utilized to identify and quantify these threats. A lot of physical phenomena for instance vibrational and rotational energy levels, phonon of biological macromolecules like DNA and protein are in the scope of THz waves frequency range which can reveal molecular interactions in food compounds generated by the formation of hydrogen bonds, van der waal’s force as well as because of hydration (8, 9). In the food sector, THz spectroscopy is used to identify antibiotics, microorganisms, toxins, additives, and hazardous substances.

In comparison to established, traditional identification methods, THz spectroscopy technique may effectively discriminate between distinct contaminated dairy products on the basis of their fatty acid profile sparing the need for sample pretreatment (10). Freshness is considered a very important parameter to adjudge the quality of pork in meat industry, by using *K*-value index. THz spectroscopy in attenuated total reflectance mode was practiced to predict the *K*-value of eighty samples of pork with a frequency resolution of 7.6 GHz, without causing any harm to its native structure. Prediction models were developed using multivariate statistical techniques including PCR’s linear regression methods and BP-non-linear ANN’s method. The results demonstrated that THz spectroscopy, in conjugation with a prediction model, can efficiently determine pork freshness. Also, it was found that the THz spectra of fresh pork lies in between 0.2∼2 THz (11). THz technology has been used successfully in the non-destructive diagnosis of toxic substances, food pathogens as well as hazardous components in food. A number of studies demonstrated that using THz in conjunction with chemometric techniques, hazardous and toxic compounds in food samples can be quickly identified and measured in even less than 5 s. The limit of detection of the approach still requires enhancement despite the most recent advancements in the quantification of toxic substances utilizing THz spectroscopy. Sometimes it may also mistakenly identify toxic samples in neutral samples even if they are absent in the samples. By utilizing time-dependent THz pulse spectrum analysis, this drawback could be eliminated (12). This technology has ingrained avid utilization, as a potent instrument with excellent detection and quantification capabilities.

### Laser induced breakdown spectroscopy

Laser induced breakdown spectroscopy (LIBS) was introduced and recommended as food analytical technique as soon as the laser was discovered in 1960. Basic principle of LIBS is based on the interaction of an intense and strong beam of laser with the food sample, causing dielectric breakdown with the generation of hot plasma. Plasma constitutes atoms, molecules, electrons, and ions with energy higher than their ground state. When these molecular species return back to their ground state, they emit specific radiations, which is further detected by a spectrometer providing identification and quantitative statistics of the elements contained in the sample (13) (Figure 1B). LIBS is commonly used to analyze a spectrum spanning the wavelength range of 190 to 850 nm.

Initially LIBS was utilized for industrial (14), space (15), cultural heritage (16), and environmental (17, 18) applications. But very recently, integration of LIBS for food authentication has drawn a lot of attention of scientific fraternity. It may be employed for the detection of adulterants and heavy metals in food products and also to ascertain the geographical origin of food (19, 20). For quick and element characterization of milk a LIBS technique was devised. To achieve precise and reliable recognition of diverse adulterated milk powders, traditional machine learning methods and convolutional neural networks (CNN) were exploited. Among the four machine learning programs (support vector machines, linear discriminant analysis, random forest (LDA), and k-nearest neighbor), combination of LIBS with CNN emerged as uncomplicated, reliable, and authentic approach for the identification of adulterants (21). In another experiment, LIBS was used to identify meat species on the basis of their element compositional differences. In the experiment, meat from different species including poultry, beef and pork were intermingled and processed into pallets and were exposed to LIBS treatment. The laser used to ablate the samples at primary wavelength of 1064 nm. Q switched mode with pulse width and rate of repetition of 4 Hz, a gate delay of 300 ns, and an integration time of 1.05 ms. Energy involved per pulse was 38 mJ. The spectra obtained were analyzed using statistical and mathematical methods. It was deduced that *R*^2^ (coefficient of determination) value and limit of detection (LOD) for chicken and pork adulterated beef were 0.999, 2.0, and 0.994, 4.4%, respectively (22). Similarly, LIBS was employed to identify milk fraud in thirteen samples both of caprine and bovine and fourteen samples ovine milk samples based on their elemental makeup. Laser operation was done in Q switched mode with rate of repetition, gate delay and an integration time of 8Hz, 650 ns and 1.05 ms, respectively. The spectra obtained from LIBS was assessed using Principal Component Analysis (PCA)to classify pure and contaminated milk samples, and partial least square regression (PLSR) was used to calculate the ratio of adulteration. The samples of milk were transformed into gel to avoid splashing and low intensity of emission. It was demonstrated from the results that *R*^2^ and LOD value for ovine and caprine milk adulterated with bovine milk were 0.996, 1.29, and 0.995, 1.9%, respectively (23). This method’s relevance for authentication purposes would grow if it had higher sensitivity for minor and trace minerals with extremely low concentrations in a complex organic matrix. Thus, it can be inferred from the foregoing discussion that LIBS can provide chemical analysis *in situ* without sample preparation, in a quasi non-destructive way, which can be employed in a variety of foods for authentication.

### Hyperspectral imaging

Hyperspectral imaging technique is fusion of spectroscopic approach with imaging which has emerged as a non-invasive identification/detection technique for food quality authentication that express prodigious spatial and spectral data for many samples simultaneously (24). Using this imaging technique the compositional and morphological characters description of a sample can be analyzed by capturing images from different spatial directions (25) (Figure 1C). Hyperspectral imaging (HSI) has certain advantages in comparison to traditional spectroscopic technologies in that complete spatial information can be drawn by this while other spectroscopic techniques including NIR provide the spectral information about an entire sample in a single spectrum, but it does not provide spatial information. HIS merge spatial and spectral details in providing the visual representation of elements inside a sample to be assessed (26, 27). This technique was first used in the 1980s using a combination of digital imaging and spectroscopy by Goetz, Vane (28). Now, this technique is widely employed in the agriculture, food, and pharmaceutical sector.

Hyperspectral images are made up of hypercubes, which are 3-D data cubes made up of many consecutive spectral bands for each spatial point of a sample (29, 30). An alike spectrum exists for each pixel displayed in an image. Each spectrum can function similarly to a biochemical fingerprint or signature allowing the recognition of chemical components of a given sample to be identified and characterized (31, 32). HIS, as opposite to NIR spectroscopy, can analyze the dispersal of multiple chemical components in a sample. This makes it possible to analyze mixed samples and identify the chemical components that make up the sample (27).

Near-infrared–hyperspectral imaging system consists of camera for image capturing, spectra analyzer, detector, illuminator and translator, all connected to a computer system (33). Whiskbroom (Diffusion of substances) imaging known as point scanner, push-broom (movement) imaging termed as linear scanner, and stare down imaging also called as wavelength scanner are three imaging modes or methods for producing a hyperspectral image of a sample from different angles (30).

The whiskbroom configuration enables hypercube retrieval by scanning every point of a sample while rotating either the sample or the detectors in the different spatial directions. This method achieves images with excellent spatial resolution, making it ideal for microstructural scanning (34). Based on the field and amplification a single image acquisition can be accomplished within 20 s using the push-broom arrangement (35). Using a 2-D detector spatial and spectral data can be collected and recorded line-by-line, making it ideal for at-line or online conveyor belt applications (36). The spectrophotometer and the sample remain at rest in the stare down position and image of the whole purview is recorded. Using one wavelength range at a time, the HSI technique collects a series of images. Using a big pile of photographs known as a hypercube, recorded images are combined to create final 3 D image (30).

Electromagnetic radiation is generally reflected, scattered, absorbed, or emitted by all biological materials. For each wavelength, different materials have prototype interaction with light. HSI is built on this framework. The discrepancies in their chemical makeup and fundamental structure are responsible for this phenomenon (37). A spectrum signature/fingerprint is a distinctive pattern representative to particular material which is created upon interaction with the light. The typical spectral signature obtained is thereby utilized to distinguish across sample kinds and classes by identifying, characterizing, and differentiating them (37). Interferences in spectroscopic data are frequently generated by scattering due to the surface heterogeneities (Williams). Therefore, before obtaining the most relevant analytical information, spectral pre-processing is usually preferred to remove particular non-chemical deviations or biasness from the obtained spectra. The data is then submitted to advanced chemometric algorithms as a process of extracting the most useful information from the data matrix and highlighting any potential discrepancies between the samples (29).

Hence, this technique has enabled the comprehensive categorization, characterization, and detection of adulterants and the best authentication approach applicable to a wide range of food products, including milk, yogurt and eggs, when used in collaboration with appropriate chemometric models. Adulteration with horsemeat in comminuted beef samples can be identified using a combination of visible near-infrared and hyperspectral imaging systems, according to Kamruzzaman, Makino (38). Using PLSR model, four wavelengths were chosen for the study: −515, 595, 650, and 880 nm. With an *R*^2^ value of 0.98, the level of horsemeat adulteration was accurately predicted. The researchers found that the HSI technology, in combination with VIS-NIR spectroscopies, can be utilized to identify adulteration in minced beef in a swift and non-destructive manner. Similarly, Zheng, Li (39) used VIS-NIR and hyperspectral imaging to detect duck meat adulteration minced lamb meat samples. The PLSR model was used to choose fourteen wavelengths, resulting in a successful model performance with an R^2^ value of 0.99. Adulteration of comminuted beef with pork can be detected using hyperspectral imaging, along with researchers reported that this technique also successfully discriminated between pure beef and pork samples from the adulterated ones (40). HIS can also be used to detect counterfeiting in honey samples and to verify the authenticity of pure honey obtained from bees, according to another study (41).

### Nuclear magnetic resonance spectroscopy

For quality management and the tracking of counterfeit labeling, nuclear magnetic resonance spectroscopy has proven to be an effective approach for authenticating foods throughout the food chain, i.e., from raw ingredients to finished products. NMR spectroscopy is quantitative in which the area of an NMR signal is directly proportional to the number of nuclei produced under specified conditions, and numerous nuclei can be employed for analysis of samples for identification and classification (42, 43) (Figure 1D). Initially, NMR applications in food were confined only to low-resolution moisture analysis; however, as technology advanced, high-resolution investigations of solid as well as liquid samples can be easily performed for structural and compositional characterization, categorization, and authentication (44) and this also aids in interactions of food components at molecular levels (45, 46). NMR analysis has excellent applicability in analyzing heterogeneous food systems, which is attributed to its non-destructive nature, high precision, and better reproducibility, as well as no special preparation of samples, is required. Jakes, Gerdova (47) research provides efficient applicability of NMR spectroscopy to identify and detect beef from horse meat. Analysis of lipid components by using low field NMR in beef and horse samples revealed enough differences to allow classification models to be developed. By reviewing the research investigations, it can be concluded that NMR has emerged as a potential tool for authentication of animal based foods.

### Raman spectroscopy

Raman spectroscopy is a non-destructive spectroscopic technique that uses light scattering to produce shifted energy frequencies. Raman signals are produced by the inelastic scattering of light from samples to be analyzed, with the consequent frequency shift revealing the stretching vibrations involved (48) (Figure 1E). The resulting spectra can be used to generate a fingerprint of a typical molecule which can be used to compare molecules in different samples and to perform structural and qualitative characterizations (49). Effortless, convenient and rapid approaches for authenticity and adulteration detection for different food categories have been developed using NIR, Raman, and, most notably, MIR spectroscopy (Table 1).

Zhao, Downey (50) conducted studies for adulteration detection in comminuted meat products with beef offals using dispersive Raman spectroscopy alone with multivariable data analysis. PLS-DA was used to evaluate the spectral data for assessing authentication of beef burger samples made according to market formulae by examining the adulteration levels in offal-adulterated and original beef burgers. With excellent accuracy, PLS-DA models correctly recognized legitimate and contaminated samples. PLS regression models also enabled the quantification of total offal and amount of extra fat in the samples. Li, Shan (51) employed Raman spectroscopy in combination with chemometrics and attempted to identify adulterants like added sweeteners viz., high fructose corn and maltose syrup in natural honey. Raman spectroscopy in combination with PLS–LDA efficiently detected the samples adulterated with high fructose corn syrup vs. authentic honey with an accuracy of 91.1% while 97.8% accuracy was detected for adulteration of honey with maltose syrup against pure honey samples. However the classification honey adulterants whether high fructose corn syrup or maltose syrup using chemometric tool PLS-LDA gave total accuracy of 84.4%.

As water seems to have a minor influence on the Raman spectrum, it can directly measure aqueous solutions, and the sample preparation is also very simple, which makes the Raman spectrum as an advanced approach for checking adulteration and authentication of milk. The use of Raman spectroscopy and chemometrics to differentiate milk powder samples resulted in chemical fingerprints for distinct types of milk powder (50, 52). A portable Raman spectrometer was used to perform online melamine measurements in milk powder. The milk powder samples were fortified with 3, 5, and 10% melamine against blank for real sample detection using Raman spectrum ranging from 450–1050 cm^–1^. Melamine had a detection limit of 0.13 percent using PLS modeling at Raman peak positions at 673 and 982 cm^–1^ (53). When Raman signal processing is improved, melamine detection can be done even if it is present in trace amounts. The presence of fluorescent molecules in the sample might affect the generated spectra which interferes the correct representation of the results. The handling of instruments is quiet sensitive as well as the interpretation of the data obtained requires the proper knowledge for the same, otherwise it might lead to the wrong interpretation of the results.

### Near-infrared- and mid-infrared spectroscopy

Vibrational spectroscopy analyzes and measures molecular and rotational vibrations caused owing to absorption of infrared light by solid, liquid, or gaseous samples (54). Vibrational spectroscopic techniques for food authentication include techniques- Near-Infrared (NIR) and Mid-Infrared Spectroscopy (MIR). Near-infrared (780–2500 nm), mid-infrared (2500–25,000 nm), and far-infrared (25,000–200,000 nm) are the three sub-regions of the IR region of the electromagnetic spectrum (1). In the MIR domain, the absorption spectra of molecules with functional groups with O–H, C–H, C = O, and N–H correspond to fundamental stretching, bends, and rotational movements (1, 55).

Infrared instruments are typically made up of the different components: a source of light, a great system for the selection of wavelength, a detector, and a signal amplifier/magnifier, which are attached to a computer system for analysis of the generated spectra (56). Even though both methods are extensively utilized, they each have their own merits and disadvantages, which help them, improve for specific applications. These approaches have the advantage of being non-destructive and generally, no sample preparation is required (56). Another benefit of NIR over MIR spectroscopy is the capability to detect reflectance. When it comes to sampling, some NIR devices allow the rotation of a sample while scanning it. As a result, NIR spectroscopy is a suitable choice for measuring heterogeneous food materials (57). MIR spectroscopy, on the other hand, has been demonstrated to perform best with small amounts and homogeneous samples, making trace material detection more challenging.

The primary vibrational bands corresponding to the functional/chemical groups present sample are measured using MIR spectroscopy (Abbas et al. (55); Esteki et al.) (1). The spectra produced by these primary bands are suitable for detecting the composition of samples and identification based on specific MIR fingerprints. On the other hand, NIR spectroscopy generates very complicated spectra. The NIR area is distinguished by broad, wide, and overlapped spectral bands. These attributes make identifying distinct chemical species difficult among different samples (Workman) (58). Another drawback of this approach seems to be that the quantitative data obtained can be influenced by a myriad of compositional or structural variables (59). Multivariate data analysis approaches have been designed to minimize extracted data from spectra generated by NIR. Chemometrics is therefore important to analyze the spectrum data and interpret it correctly. Preprocessing techniques aids in removing the unwanted variations or technical conformations from raw data which can further be improved by using chemometric tools.

Pure chopped beef was differentiated from those contaminated with organ meats (kidney and liver) using MIR spectroscopy in combination with chemometrics where the SIMCA model differentiated pure and adulterated beef with 92% accuracy (Al-Jowder et al.) (60). The findings also revealed that prediction models based on the combination partial least squares (PLS)/CVA approach can efficiently identify between beef and offal categories. At a concentration of around 10% (w/w), the authors were able to identify and characterize these adulterants, although the level of adulteration was still fairly significant. Finally, PLS regression models were used to calculate the number of offals.

Visible-near-infrared (Vis-NIR) spectroscopy was used by Rady and Adedeji to identify plant and animal proteins as adulterants in minced beef and pork. Adulterant classification, prediction, and wavelength selection were explored using a variety of chemometric approaches. The samples were separated into three levels of classification. In the first classification level, the samples of beef pork, chicken, texturized vegetable protein, and gluten were classified as pure or adulterated. The statistical models used for classification successfully distinguished between the pure (100%) and adulterated samples (96%). Further in the types of adulterants were detected during second level of classification. Individual adulterant detection was less successful, with categorization rates ranging from 69 to 100 percent (61). In the third level of classification, quantitative classification of adulterants was done by applying different regression models. The PLS regression models precisely quantified the adulterants detected in meat samples. Therefore, Rady and Adedeji resolved by this investigation that Vis-NIR spectroscopy has potential to examine authentic minced meat products quickly and reliably. For the identification and quantification of pig adulteration in beef meatballs, Kuswandi et al. (62) used chemometrics for analyzing NIR spectral data obtained for examining adulterated pork. For quantitative analysis, the PLS regression model was employed, while LDA model was used for qualitative estimation. The LDA models precisely identified pork adulterated beef meatball samples based on first-derivative spectra. The PLS and LDA models also produced findings that were quite similar to the immuno-chromatographic approach employed as a control. The procedure proved to be a quick and effective way to verify the halal status and detect pork in beef meatballs.

### Fourier transform spectroscopy

The Fourier transform spectroscopic technique is categorized under vibrational spectroscopy that employs interferometers to modulate the Fourier transform algorithm in the form of an electromagnetic signal to transform sample data into an optical spectrum obtained on a computer system (63). In the interferometer, light beams are scattered and then reassembled. Due to the simultaneous measuring of wavelengths, this approach is quick and has a better signal-to-noise ratio. Fourier Transform can be also used in optical, infrared, nuclear magnetic resonance, Raman, electron spin resonance spectroscopies as well as mass spectrometry (63). However, this method is generally applied to infrared spectroscopy.

This spectroscopic technique is successfully utilized for detecting and characterization of different types of adulterants in meat and processed meat products (64, 65). Meza-Márquez, Gallardo-Velázquez (64) interrogated the presence adulterants used in processed beef adulteration with different adulterants- horsemeat, trimmings of beef, and texturized plant protein by using mid-Fourier transform infrared (MID–FTIR) spectroscopy and chemometrics. Principal component analysis (PCA) and PLS regression models were used to detect and quantify the adulterants within the minced meat. And absorbance spectra had an excellent correlation with the concentration of constituents and the percentage of adulteration was measured. 100% accuracy of classification strived through developed models (64).

Schmutzler, Beganovic (66) developed and compared three different setups based upon FT-NIR in combination with chemometric models to detect the adulteration of veal sausage with pork meat. The results revealed that the application of FTIR along with chemometric models successfully distinguished between pure and adulterated samples (66). Alamprese, Amigo (65) measured the FT-NIR spectra of 198 samples of raw, frozen-thawed, and cooked beef and turkey meat with an aim to identify and quantify turkey meat adulteration in fresh and processed beef samples. The PLS regression models worked efficiently and accurately distinguished between modest and higher levels of adulteration. Alamprese and Casiraghi (67) studied the potential of employing FT-IR spectroscopy as a quick and easy way to authenticate Atlantic mullet (165 samples) and flounder (134 samples) when replaced with red mullet (132 samples) and plaice (124 samples). They also examine the efficacy of spectroscopic techniques to distinguish between fresh and frozen-thawed Atlantic mullet filets. With high sensitivity (>70%) and specificity (100%), LDA enables Atlantic mullet to easily classified from expensive red mullet.

### UV–Vis and fluorescence spectroscopy

UV–Vis spectroscopy is among the most extensively used food analytical approach involving measurements pertaining amount of light absorbed by the samples. The ease of handling, the potential to determine an extensive array of compounds, non-destructive nature, high precision, better reproducibility, and modest discernment are the key benefits of this spectroscopy. Beer’s law, which provides the basis of this approach, states that the amount of an analyte dissolved in a solvent is directly proportional to the amount of light absorbed.

Fluorescence is a process of emission of light by fluorophores or fluorescent compounds after UV or VIS light has been absorbed. Fluorophores are compounds that have more than one conjugated bond bearing aromatic rings and linear structures. The sample illumination geometry and optical density have a big impact on the apparent fluorescence intensity and spectrum pattern. The right-angle observation of centrally illuminated cuvette is the most frequent geometry employed in fluorescence spectroscopy. However, right-angle measurements are difficult to make when viscous samples are to be analyzed. Another issue with this technique is that when handling biological items such as meat or seafood, the optical characteristics may reduce the intensity of fluorescence with increment in path and reabsorption (68, 69). To address these issues, front-face fluorescence spectroscopy can be adopted, which results in very little fluorescence spectral distortion during both emission and absorption. The excitation light is focused on the front surface of the sample, and the fluorescence emission is collected from the same area at an angle that minimizes reflected and scattered light. This method works well with concentrated, opaque, or solid samples (70, 71).

The ability of fluorescence spectroscopy to distinguish between fresh and frozen-thawed samples of sea bass (*Dicentrarchus labrax*) was assessed (72). Four different fluorophores used were NADH (340 nm), tryptophan (290 nm), riboflavin (380 nm), and vitamin A (410 nm). The findings revealed that the approach could distinguish not only between fresh and frozen-thawed sea bass samples but the variations in the quality of different samples of frozen fish before storing. Factorial discriminant analysis (FDA) applied to the spectra recorded after excitation sets at 340 and 380 nm, 72 samples out of total 78 were correctly classified. Thus, it can be concluded that fluorescence spectroscopy along with chemometric tools could be a reliable method to discriminate fresh and thawed samples.

Different chemical and rheological factors were used to successfully discriminate distinct beef muscles using fluorescence spectroscopy (73). Seven bovine muscles were distinguished using fluorescence spectroscopy in the range of 305–700 nm to generate emission spectra, achieved by adapting the excitation wavelength at 290, 322, and 382 nm. Fluorescent spectra were used to categorize different muscles based on different parameters viz., fat, protein, collagen, and dry matter content.

Fluorescence spectroscopy has a similar potential for the sorting and depiction of beef muscles (66 samples)-Semitendinosus (*n* = 24), Rectus abdominis (*n* = 24), and Infraspinatus (*n* = 18), using fluorescence spectroscopy followed by data treatment using PLS-DA and PLS regression models. Emission spectra were recorded in the range of 305–400, 340–540, and 410–700 nm by fixing the excitation wavelength at 290, 322, and 382 nm, respectively. Excellent classification (100%) was obtained for three muscles. Discriminant analysis applied on emission spectra in the range of 410–700 and 340–540 nm gave 96 and 90% of good classification respectively for the three muscles. The findings of the investigation concluded fluorescence spectroscopy combined with chemometrics has a lot of potential for identifying distinct beef muscles (74). Akin observations were subsequently achieved by (75), indicating the capability of this spectroscopy to perform muscle sample discrimination. There are few studies that have used this spectroscopic technique to differentiate fresh and frozen-thawed meat. Ruoff, Luginbühl (76) also used front-face fluorescence spectroscopy to authenticate unifloral and multi floral honey varieties that had earlier been categorized using traditional approaches like chemical, pollen, and sensory analyses. PCA and LDA were used to analyze spectral data. Front-face fluorescence spectroscopy was found to be successful in authenticating the origin of honey as well as distinguishing ethnic origins within the similar unifloral honey variety.

## Applications for authentication and spuriousness of animal foods

### Meat and poultry

Meat authenticity knowledge and understanding have grown in recent years, and numerous analytical methods have been presented and validated in the struggle against meat fraud. Although these technologies have demonstrated the ability to reliably detect minimal concentrations of adulterants, these methods are disparaging, tedious, expensive, and labor-intensive. As a result, these are unsuitable for quick and fast detection, especially in the meat industry’s incredibly quick manufacturing and processing environment. However, as the food business strives toward non-destructive, non-invasive, easy, and online approaches, contemporary analytical technologies may improve this process. Adulterating foods has a huge commercial advantage because of the surge in manufacturing and processing of food to serve the increasing population. Adulterating foods also cause deception and may jeopardize food safety. Food fraud is frequently found in high-value products like beef. Meat and meat products are an important part of human nutrition (87). Meat is high in biologically important owing to the presence of an adequate amount of proteins, fats, B-complex vitamins, and minerals including zinc and iron. All these nutrients mentioned are required for normal physiological processes and the growth of humans (88, 89).

Since meat is generally sold as fresh and processed at the domestic level, it was rarely associated with adulteration (90, 91). Meat consumption preferences have shifted recently more toward processed and ready-to-cook/eat convenient foods like comminuted meat, meatballs, sausages, and meatloaves (90, 92). When these items are prepared, the morphological properties of the flesh are lost which makes it quite grim to distinguish between different muscle types, sources, and species. This provides the means to the processors or manufacturers to falsely alter or substitute premium-quality meat with low and cheap quality muscle types or species (64, 65). Customers suffer the consequences of these types of fraudulent activities by losing money, the ingestion of meat that is forbidden in some religions, unintentional exposure to common allergens, and compromise food safety (93).

As a result of these fraudulent actions, meat products must be closely monitored, controlled, and inspected during manufacturing, storage, and distribution (94). As a result, numerous analytical approaches have been presented and subsequently assessed in the struggle against meat fraud (95, 96). To identify meat species, many analytical approaches are used, most of which are based on protein or DNA based assays and polymerase chain reaction (PCR and real-time PCR) (97, 98). Protein-based techniques, such as electrophoretic and immunological techniques viz., enzyme-linked immunosorbent assay (ELISA), can be inadequate in distinguishing closely related meat species (29). Methods such as electrophoresis and chromatography (29, 99, 100) have also showed potential in this regard. While processing in a meat production plant, these methods are often used to examine, investigate, and quality analysis of meat and poultry products based on eminence and reliability factors (94). These authentication techniques, while trustworthy, specific, and sensitive, have several limitations, including the fact that these are time-consuming, tedious, costly, as well as complex laboratory requirements, sensitive and careful handling of sophisticated procedures making them subject to subjectivity. As a result, traditional approaches are ineffective for rapid analysis and quick identification, especially in the modern meat industry’s fast track of manufacturing and processing of products.

Quick, non-destructive, non-invasive, precise, efficient, and more reproducible analytical methodologies for authenticity and fraud identification in meat and poultry products have been developed due to the general shortcomings of traditional methodologies (37, 101). Fourier transform infrared spectroscopy (64, 65, 102) color and X-ray imaging (103, 104), near-infrared spectroscopy (61, 105), hyperspectral imaging (39, 106), and Raman spectroscopy (102) have now been explored for use in the authentication and/or identification of adulterants in processed meat products.

Egg quality and freshness are now frequently assessed using spectroscopic techniques. Because these procedures are non-destructive and provide precision, accuracy, speed, and rapid as well as reproducible results. Spectroscopic techniques promote the integration of online egg shell grading applications, with apparent benefits for both manufacturers and consumers. Applications of spectroscopy technologies such as VIS-NIR, NIR, Raman, microwaves, hyperspectral imaging, and pulsed IR thermography for non-destructive evaluation of shell egg quality and freshness, to encourage research in this sector and provide some directions for fulfilling business needs (107).

Utilization and employing of non-destructive technologies is critical for the meat processing industry to undertake safety and authenticity assessments of different types of meat types and products without altering the characteristics of the original product. Surface examination of samples without employing any invasive or disruptive techniques that might affect the quality characteristics of food is termed a non-destructive analysis. The primary benefit of non-destructive spectroscopic approach application is that it may measure the physical and chemical characteristics of foods without affecting the sample taken for analysis. Hence, the samples can be employed for authenticity check in the entire processing and distribution chain using non-destructive procedures, resulting in no product losses. The meat industry lacks fast and easy, non-destructive, and non-invasive procedures for the authentication of processed meat products that can be used *in situ*, this manuscript attempted to compile the most recent spectroscopic techniques that have the feasibility to meet meat product authentication needs. Table 2 depicts a comprehensive review of different research studies about applications of various spectroscopic techniques used for checking adulteration and authentication of various meat, meat-based and poultry-based food products.

**TABLE 2:** Spectroscopic techniques for authentication/adulteration of meat, poultry and seafood.

Type of animal origin food	Issues related to authentication	Spectroscopic technique analysis	Data Analysis/Chemometrics	Experiment conditions	Outcomes	References
***Seafood***						
Fish filet samples	Classification of deep-frozen fish filets	Raman Spectroscopy (wavelength of 532 nm)	Sensitive non-linear iterative peak-clipping algorithm (SNIP)	Twelve samples of fishes, spectra recorded in range from 300–3400cm^–1^ The scattered light detected through CCD camera operated at 220 K. Acquisition time/spectrum:10s	Efficient classification. Proven to be a potential screening tool in fish filet identification The level of accuracy of the obtained spectra was 95.8% with 92 out of 96 spectra were accurately allocated to the respective sub clusters of the species	(127)
Salmon filets	Detect variation in fish muscle in terms of total variable counts, specific spoilage organism, or any other changes in composition during storage for different combinations of storage time, temperature, and packaging atmosphere	Fourier transform infrared spectroscopy (FTIR)	Principal component analysis (PCA) and partial least square regression (PLS-R)	Salmon filets (3*4*1cm) weighing 20 g were stored under three conditions: air packaging, modified packaging 50% N_2_/40% CO_2_/10% O_2_ with lemon juice, 50% N_2_/40% CO_2_/10% O_2_ without lemon juice.	FTIR spectra with PLS-R allowed bacterial load estimation. Lemon juice with modified atmosphere packaging pronouncedly reduced *Brochothrix thermosphacta* and H_2_S producer counts Regression coefficient value was 0.88 and 0.90 for *Brochothrix thermosphacta* and H_2_S producer	(128)
Bighead carp (*Aristichthys nobilis*)	To determine the freshness	Near-infrared reflectance spectroscopy (NIRS)	Partial least-squares regression (PLSR) in combination with Competitive adaptive reweighted sampling (CARS)	150 samples, Spectral range:1000–1799 nm in reflectance mode	Freshness prediction models were successfully developed with satisfactory high coefficients of prediction for different freshness indicators like pH, total volatile basic nitrogen (TVB-N), thiobarbituric acid reactive substances (TBARS), and ATP-related compounds (*K*-value) Coefficients of prediction (Rp) of 0.945, 0.932, 0.954, and 0.807, respectively	(129)
Shelled shrimp (*Metapenaeus ensis*)	To distinguish between fresh, frozen samples of shelled shrimp, to check adulteration and mislabeling	VIS-NIR (400–1000 nm) in paired with a hyperspectral imaging system	Discrimination Random forest and soft independent modeling of class analogy	Fresh (*n* = 79 samples), low temperature stored (*n* = 80 samples), frozen (*n* = 65 samples) as well as thawed samples (*n* = 80 samples) of shelled shrimp	Satisfying results were derived with accurate classification rates of 91.11 and 88.89% for both models	(124)
Green Lipped mussel (*Perna viridis*) and Japanese jack mackerel (*Trachurus japonicus*)	To assess the microplastics in seafood models	Raman Spectroscopy	Automated Raman Mapping approach	Mussel shell was thawed for soft tissue extraction, mackerel was cut into pieces with bones prior to digestion. The both samples were digested at 40C for 48husing KOH, KOH with H_2_O_2_, KOH, EDTA and H_2_O_2_,	Polypropylene, polyethylene, poly (ethylene terephthalate), and polystyrene were identified as microplastics in fragmented and fibers Form The outcomes were deemed adequate for identifying microplastics larger than 30 μm	(130)
Fish	To identify fish species and their substitution	Ultraviolet-visible (UV-Vis) spectroscopy	Principal Component Analysis (PCA)	Sixty fish samples from 12 commonly consumed fishes species. Scan range from 200–400 nm	Successful identification and genetic evaluation of fish species	(111)
Salmon	To identify the wild and farmed salmon	Direct Analysis in Real-Time (DART) coupled with High-Resolution Mass Spectroscopy (HRMS)	Principle Component Analysis (PCA)	26 wild salmon from Canada and a total of 74 farmed salmon, arising from aquaculture plants of Canada (25), Norway (25) and Chile (24), all of *Salmo salar* species,	PCA showed a clear distinction between wild and farmed salmon, which accounted for the explanation of 99.38% of variance	(131)
European sea bass (*Dicentrarhuslabrax*)	Authentication of proper labeling issues for European sea bass as per International labeling regulation	Inductively coupled plasma atomic emission spectrometer for macro-, micro-and toxic elements detection	Principal component analysis (PCA) and Sample classification through discriminant analysis	Samples were collected from 18 different Italian and foreign sources out of which 45 were wild, 85 were intensively reared, 20 were semi-intensively farmed, 10 extensively farmed	Elemental composition and toxic elemental detection helped in checking regulations limits. Using fatty acid profiles, accurate subject allocations based on production technique, origin, and stocking density were shown with high prediction rates of 94, 92, and 92%, respectively	(132)
Fish and shrimps	Detection of brevetoxin B (BTX)	Spectroscopic ellipsometry (SE) and attenuated internal reflection spectroscopic ellipsometry (TIRE)	two anti BTX aptamers using predictive modeling tools and an exclusion method	Sensors capable of detecting BTX ranging from 0.05–1600 nm in TIRE and 0.5–2000 nm in SE configuration	Successful detection of BTX toxins with detection limits of 1.32 ng/ml for SE and 0.72 ng/ml for TIRE configurations	(133)
Caviar	To distinguish between Aquitaine caviar and other caviars sample.	^1^H-NMR spectroscopy	Multivariate models-Soft Independent Modeling by Class Analogy (SIMCA) and orthogonal partial least square discriminant Analysis	91 aqueous extracts of caviar samples, NIR parameters: a 500-ms acquisition time, an 80-ms mixing time, an 8090-Hz spectral width, and a 1.5-s relaxation delay.	NMR metabolic profile provided the freshness estimation as well as the shelf life of caviar cans along with the characterization of different metabolites Sensitivity and specificity of the 2-year SIMCA model were reported to be 0.94 and 0.65, respectively	(134)
Atlantic salmon	For quick identification of rainbow trout adulteration in Atlantic salmon	Combination of Raman Spectroscopy with a machine learning approach	Pre-processing methods-first and second derivative, multiple scattering correction. Recursive feature elimination, genetic algorithm, and simulated annealing and supervised K-means clustering algorithm.	Adulterated samples contained different concentrations (0–100% w/w at 10% intervals) of rainbow trout mixed into *Atlantic salmon*. Spectral analysis was done in range of 500–2000 cm^–1^	The developed model of GA-KM-cubist machine learning with Raman spectroscopy was effective in the adulteration detection of Atlantic salmon Determination coefficient (*R*^2^) was reported to be 0,87 Root mean square error of prediction sets (RMSEP) was 10.93	(135)
King Salmon Atlantic salmon and Rainbow trout	To study lipidomics properties for better distinction among these three salmonids	Hydrophilic interaction chromatography-mass spectroscopy	LIPID MAPS prediction tool, One-way analysis of variance, principal component analysis	15 samples (05 for each King Salmon Atlantic salmon and Rainbow trout), MS parameters: ion spray voltage-4500V, ion spray temperature-500°C, ion drying gas pressure 24 psi, nebulizer gas pressure- 30 psi, curtain gas pressure-25 psi declustering potential-75V, collision energy-40V	Phospholipids of m/z 802.8 and m/z 834.8 were reported to be potential markers for species identification	(136)
Different Fish and seafood Samples	To develop a protocol for rapid authentication of seafood	MALDI TOF Mass Spectrophotometer	R studio 1.1.419 software, Flex analysis 3.4 software		Distinguish different seafood species on the analysis of muscle tissue processed by the acid method.	(118)
***Meat and poultry products***						
Minced Beef	Adulteration with pork and duck meat	Near-infrared spectroscopy (12,500–5400cm^–1^) With average of 64 scans with 16cm^–1^ Sample preparation for NIR analysis-2 g of each sample in glass vial of 1.2 cm in diameter and 2.0 mm in wall thickness with dense packing	Discriminant analysis and Partial least squares	For pork adulteration in beef samples- 10–80% (w/w) with 10% increment were blended with beef to get 72 (3 × 3 × 8) blended samples along with 3 pure beef meat and 3 pure pork meat samples. For duck and pork adulteration- 5–40% for pork and 5–40% for duck with 5% increment replaced with beef. Total 64 adulterated (2 × 2 × 2 × 8) along with 18 pure samples (each of beef, pork and duck was six) were prepared.	Discriminant analysis provided best results with classification rate of 100% for binary system and 91.5% for ternary systems within selected wavelength. Optimal PLS models predicted adulterant levels with correlation coefficient of 95.80 and 95.69%.	(137)
Mutton and beef	Adulteration with pork meat or mutton	Fourier transform infrared spectroscopy (4000–450 cm^–1^ at resolution of 0.4 cm^–1^)	Partial least square discriminant analysis and support vector machine	180 samples Sample were sliced and defatted, dried in an oven for 24 h. Then ground to uniform size and stored at 4°C till further analysis.	In PLS-DA model, coefficient of determination for calibration and testing sets was 0.99 with RMSEC 0.06, and RMSCV and RMSEP with value 0.08, predicting 100% model accuracy.	(138)
Fish meal, poultry, porcine, bovine and ovine samples	To distinguish different sources of animal originated feed samples based on specific lipid characteristics	FT- Raman Spectroscopy (3600–400 cm^–1^ at resolution of 4 cm^–1^ With 128 scans per each spectrum Laser power- 450 mW)	Principal component analysis and partial least squares-discriminant analysis	105 processed animal-derived feedstuff samples [29 fishmeal and 76 meat and bone meal (25 from poultry, 23 from porcine, 14 from bovine and 14 from ovine sources)] Sample preparation- 8 g sample were milled to pass through 1 mm screen for lipid extraction.	Special peak ratios of 1645/1748 and 1645/1445 with high correlation *r*^2^ > 0.94 with high degree of unsaturated fatty acids	(139)
Beef and Pork	Detection of minced beef	Multispectral imaging spectroscopy	Partial least square discriminant analysis (PLS-DA) and linear discriminant analysis (LDA)	220 meat samples Adulterated samples were prepared ranging from 10–90% with 10% increment. Samples were analyzed at 18 different non-uniformly distributed different wavelength (405, 430, 450, 470, 505, 565, 590, 630, 645, 660, 850, 870, 890, 910, 920, 940, 950, and 970 nm)	98.48% overall correct classification was achieved to distinguish between pure and adulterated samples for PLS-DA and LDA. While for independent testing of pure and adulterated samples, PLS-DA was more accurate than LDA Limit of detection was 10% adulteration of pork in beef and vice versa	(40)
Scallop, shrimp, pig liver, chicken, beef and mixed sample	Identification of different meat species	Laser-induced breakdown spectroscopy	Multiplicative scatter correction (MSC) and K-nearest neighbor (KNN) model	6 samples were prepared into pellet form with diameter-40 mm under pressure of 20MPa. LIBS experimental setup YAG Laser (wavelength- 532 nm; repetition rate- 10 Hz; pulse width- 8 ns; pulse energy- 30 mJ) Emission spectral range- 200–950 nm, resolution- λ/Δλ = 5000	Recognition rate enhanced from 94.17 to 100%, while decline in prediction coefficient of variance from 5.16 to 0.56% was revealed proving MSC and LIBS to be accurate and stable in meat species authentication.	(140)
Poultry –processed products	Adulteration detection	Liquid chromatography-mass spectroscopy	LC-QQQ multiple reaction monitoring (MRM) method	12 different processed poultry based products samples	Resolves the purpose of product quality monitoring, check on food composition, compliance with declared labeling and detection of fraudulent practices.	(141)
Fresh meat	Adulterated with different types of beef and pork offals	Vibrational spectroscopy (wavelength range- 1800–1000 cm^–1^) and Fourier transform infrared (FTIR) spectroscopy wavelength range- 4000–550 cm^–1^	SIMCA, LDA	Samples comprise of three categories -Three types of beef meat cuts (beef ribeye, beef flank steak, beef chuck steak) - Three types of pork offal (pork heart, pork liver, pork kidney) - Three types of beef offal (beef liver, beef omasum and beef honey comb tripe) 90 adulterated samples were prepared by replacing 10, 25, 33.3, 50, and 66.6% beef meat by offals	SIMCA model proved to be best for beef offals identification while LDA for pork offals using non-scaled spectra More than 99% accuracy in detecting adulteration Could determinate the type of offal in the sample with >80% confidence, and to quantify five types of offal in an accurate manner (*R*^2^ > 0.81)	(142)
Eggs shells	To determine egg freshness through external scanning of egg shell	Raman spectroscopy	Partial least square regression model of 100–3000 cm^–1^	125 samples, Raman spectroscopy parameters, the acquisition band −100–3000 cm^–1^; resolution- 12 cm^–1^; integration time −5 s; number of scans—3 times; and detection distance between the probe and the egg shell surface—6 mm	More than 0.9 value of correlation coefficients was observed with Haugh unit, albumen pH and air chamber diameter, while 0.8 value for air chamber height, indicating strong relation of Raman spectrum of egg shell with freshness.	(143)
Eggs	identify fake and poor quality eggs	Raman spectroscopy (1800–600 cm^–1^) and Raman hyperspectral imaging (1500–390 cm^–1^)	Principal component analysis, Partial least squares discriminant analysis, multiplicative scatter correction	Samples were divided into two groups: one group-real chicken eggs and other group of fake eggs. Raman hyperspectral imaging revealed that fake eggs exhibit more-intense chemical images at an optimal waveband centered around 1295 cm^–1^	Raman techniques identify the fake eggs as the chemicals used in manufacturing of fake eggs and provide 100% accuracy.	(144)
Eggs	To verify the authenticity of native eggs	Near infrared spectroscopy	Data driven-based class modeling DDCM	122 egg samples of three types one-native (*n* = 38 samples and while other two as feed eggs, *n* = 24 samples each),spectral range varied from 10,000–4000 cm^–1^ with a resolution of 3.856 cm^–1^ interval,	NIR spectroscopy on combination with class-modeling is efficient tool for authentication of a specific type of native eggs	(145)
Eggs	Screening and sorting of organic eggs	^1^H NMR spectroscopy	Principal component analysis followed by linear discrimination analysis (PCA-LDA) and Monte-Carlo cross validation	344 samples of chicken eggs, out of that 214 were barn/free-range eggs while 130 eggs were from organic farms. Separated egg white and yolk from different were freeze dried for further analyses. NMR spectra were acquired at 290 K with relaxation delay for 3 s, and acquisition time of 11 s.	93% accurate recognition/identification of the organic eggs was evaluated on employing NMR spectroscopy with chemometrics	(146)
Beef muscles	To distinguish three beef muscles (*Longissimus thoracis, Rectus abdominis and Semitendinosus*)	Classical front face (FFFS) and Synchronous (SFS) Fluorescence spectroscopy	Partial Least Square Discriminant Analysis (PLSDA), Support Vector Machine associated with PLS (PLS-SVM) and Principal Components Analysis (PCA-SVM)	261 samples of three beef muscles- *Longissimus thoracis-139 samples, Rectus abdominis-58 samples and Semitendinosus-64 samples*. 100 g of each were collected at 24 h post mortem. 2610 total emission spectra recorded (261 muscles × 2 repetitions × 5 excitation wavelengths).	For the FFFS, the PLS-SVM with the 382 nm excitation wavelength gave the best identification results. For SFS, when performing discrimination of the three muscles, the 120 nm gave best results	(75)
Minced beef and horse meat	Detection of minced beef adulteration with horsemeat	Multispectral imaging spectroscopy	Partial least square discriminant analysis, random forest and support vector machines	110 samples, four levels of adulteration, 20–80, 40–60, 60–40, and 80–20% (w/w) containing each meat samples, multispectral images were acquired in 18 wavelengths	SVM model gave 95.31% overall correct classification for independent batch validation and correct classification of fresh and pure ground meat samples	(147)
Beef Steak	To differentiate between grass-fed and grain-fed beef	Near infrared reflectance (NIR) and Raman spectroscopy	Partial least squares discriminant analysis (PLS-DA) and linear discriminant analysis (LDA)	Total 108 beef steak samples classified as grass-fed (*n* = 54) and grain-fed (*n* = 54) beef steaks	The NIR spectra accurately discriminated between grass- and grain-fed beef on both fat (91.7%, *n* = 92) and lean (88.5%, *n* = 96), as did Raman (fat 95.2%, *n* = 82)	(148)

### Seafood

Seafood fraud is quite a difficult task to combat due to the ongoing evolution of fraudulent tactics and its consequences for both consumers as well as worldwide trade (108, 109). As a result, it is indeed critical to keep an eye on the entire food chain to spot and avoid fraud, such as species swaps, which is the most prevalent deception in the seafood sector (110). With the industrial advancements in the food sector, quick, simple, and reliable solutions for food authenticity checks are always sought in the entire food supply chain. The quest for excellent quality and safety both during fish farming and processing implies stringent process control, monitoring, and quality assurance standards. Fish and fish products are easily and regularly prone to fraud tactics due to massive international production, imports, and consumption. Species swapping, terrestrial origin misrepresentation, manufacturing process or aquaculture falsification, and replacing fresh with frozen/thawed product exchange are all instances of fish and seafood product authenticity challenges. As a result, meeting this demand necessitates the use of appropriate analytical instruments both before and after food processing and production. Quick, efficient, simple, user friendly, limited or nil sample preparation, and a very small amount or no wastage sample are all desirable aspects of such technologies and these features are peculiar characteristics of different spectroscopic technologies including mid-infrared, near-infrared and visible spectroscopy (111).

Ghidini, Varrà (105) described qualitative spectroscopy in combination with chemometrics in seafood products. Surimi from marine fish was used to detect and recognize using near-infrared diffuse reflectance spectroscopy (112). Sixty-four samples of six different species of fishes, including Atlantic salmon (*Salmo salar*), European anchovy (*Engraulis encrasicolus*),bluefish (*Pomatomus saltatrix*),horse mackerel (*Trachurus trachurus*), red mullet (*Mullus surmuletus*), flying gurnard (*Trigla lucerna*), were analyzed using Raman spectroscopy combined with chemometrics to distinguish between fresh and frozen/thawed samples of fish (113). Various research studies investigated and reported that sophisticated spectroscopic techniques effectively resolved authenticity problems relating to fish and seafood counterfeiting. Using matrix-assisted laser desorption/ionization time-of-flight mass spectrometry (MALDI-TOF MS) and high-performance liquid chromatography-mass spectrometry (HPLC-MS/MS), a recent study examined the variations in exudates of fish muscle when subjected to different varying freeze and thaw cycles and along with different frozen storage periods. The results indicated that the developed method can be a promising strategy to evaluate the change in fish muscle or other animal muscle-based foods (114). In recent years, mass spectrometric, metabolomic, and chemometric techniques have been used to investigate different fish for taxonomic classification, authentication, and quality assessment (115–117). The pre-treatment of the fish muscle samples before subjecting to spectroscopic analysis was reported to increase in effectiveness and efficacy of analysis and hence better reproducible results (118).

The key morphological traits of seafood that allow the buyer to recognize one variety from another are usually obliterated during processing, which is a significant challenge to authenticity in seafood processing. In the seafood sector, species replacement is a very widespread kind of economic adulteration (119, 120). In crabmeat samples, Gayo and colleagues used visible (VIS) and near-infrared (NIR) spectroscopy to identify and quantify species authentication as well as monitor adulteration (31). In another study, the issue of the mixing of Atlantic blue (*Callinectes sapidus*) crabmeat with blue swimmer (*Portunus armatus*) crab was explored and studied using spectroscopic techniques. The scientists discovered that the predominant features in the crabmeat spectra were dominated by water absorption bands, with a decline in sample absorbance as the percentage of adulteration increased (31, 121). For samples containing blue swimmer crabmeat, VIS/NIR spectroscopy was able to identify species authenticity and economic adulteration to less than 6%. Furthermore, these authors reported using multiple data pre-treatments in combination with NIR spectra, including moving average, a combination of first and second derivatives, and multiplicative scatter correction, before creating PLS regression models (31, 122). Gayo and his colleagues discovered that chemometric methods paired with the visible and near-infrared spectroscopic techniques were effective in detecting and assessing species’ originality and sullying in crabmeat. In this study, a surimi-based crab meat mimic was used to taint crab meat samples from the Atlantic blue (*Callinectes sapidus*) and blue swimmer (*Portunus armatus*). The scientists discovered that both PLS and principal component (PCR) regression techniques gave the best way of detecting counterfeiting in crab meat samples falsified with surimi after pre-processing the spectra using the first derivative. However, samples with less than 20% adulteration cannot be estimated accurately by this approach (31, 121, 122). NIR spectroscopy paired with PCA was used by Brodersen and Bremner (123) to distinguish between shrimp (*Pandalus borealis*) and a commercial freezer trawler. Before being steam-cooked and skinned, the shrimp were cooled, preserved in freshwater, and brine solution of varied concentrations (123). The researchers demonstrated that using chemometrics and NIR spectra recorded from differently pretreated whole shrimp and minced fresh samples, could detect and distinguish between frozen and thawed material samples, whole or minced shrimp, along with salt level, flesh pH, cooking time as well as temperature (123).

Qu, Cheng (124) used VIS-NIR spectroscopy in conjunction with a hyperspectral imaging technique to distinguish between fresh (*n* = 79 samples), low temperature stored (*n* = 80 samples), frozen (*n* = 65 samples) as well as thawed samples (*n* = 80 samples) of shelled shrimp (*Metapenaeus ensis*). By condensing the 381 wavelengths to 08 ideal ones, the calculation burden was significantly minimalized that is 75.56–63.33% by Random forest (RF) and 95.56–86.67% by soft independent modeling of class analogy (SIMCA). The findings of this research showed that combining chemometrics and spectroscopy was effective in detecting illegally replaced and mislabeled commodities (124). The authors validated the use of VIS-NIR spectroscopy along with chemometric tools – RF method and SIMCA in classifying fresh shrimp from cold storage or frozen shrimps with the classification rate of ninety-one percent and eighty-eight percent, respectively (124). Wu and companions also used hyperspectral imaging in conjunction with chemometric methods to analyze the impeccability and reproducibility of detection of gelatin adulteration in prawns (125).

The feasibility of NIR spectroscopy as a method of authentication of wild European sea bass (*Dicentrarchus labrax*) was studied by Ottavian, Facco (126). The effectiveness of three distinct chemometric approaches was investigated for NIR spectral analysis and their capability to distinguish wild and farmed samples of sea bass was assessed by the author. The data collection included 66 validation samples (32 declared wild and 34 declared farmed) with declared production techniques and 38 calibration samples with determined attribution of production method. The three chemometric strategies- partial least-squares discriminant analysis (PLS-DA) and the wavelet-based WPTER (wavelet packet transform for efficient pattern recognition) method and principal component analysis, revealed that near-infrared spectroscopy may also be used to reliably distinguish between wild and farmed sea bass, with classification performance comparable to chemical characteristics and morphometric attributes. Furthermore, in comparison to traditional and conventional methods for classification and identification, NIR-based categorization methods were reported to be comparatively easier, quick, cheaper, and environmentally safe along with this method does not have any chemical requirements too (126). Table 2 depicts a brief review of different recent research studies about exploitation of various spectroscopic techniques employed for assessing adulteration and authentication of sea foods.

### Milk and milk products

Worldwide, milk is contemplated as the most significant and nutrient-dense food, consumed in its natural form or in the form of dairy products. Owing to growing demand of milk and milk products as well as an increase in its consumption pattern these products are more liable for food fraudulent practices (149). A large number of frauds pertaining to partial replacement of milk fat or protein, swapping of high value milk from one species with a lower valued milk from other species (150), addition of adulterants (151), presence of undeclared ingredients in the products, false information regarding the geographical origin (152) etc., have been reported in literature. Furthermore, the mixing of milk obtained from non-declared species could pose health hazards due to allergies, as well as ethical connotations on account of religious beliefs or individual preferences prohibiting the consumption of milk from certain specific species (153). Recognition and confirmation of species is of paramount significance in expensive tradition milk commodities like cheese marked with logos of European Union (EU)- PGI (protected geographical indication), PDO (protected designation of origin) and TSG (Traditional specialty guaranteed). Because of higher economical and nutritive excellence, aroma, textural attributes, these products are more susceptible to adulteration practices (154). Use of FTIR spectroscopy was advocated for detection and quantification of the proportion of adulteration of cow milk with different ratios of milk of goat and sheep (155).

Moreover, with the advent of technology and increasing awareness among the consumer, ethical economic and health challenges, there arises an urgency for simple, stringent, precise, rapid, innovative, green and non- destructive quality control techniques for identifying the geographical origin, detection of adulterants and fraud in milk and milk products (156). Authentication of milk based commodities entails using analytical techniques to verify whether the products confirms to the specified labeling and complies with applicable laws and regulations (157). As discussed in the previous sections, various comprehensive and focused approaches, including as molecular, chemical, protein-based, and chromatographic techniques, have traditionally been used to identify the species, origin, unusual ingredients, and type of processing procedure used to manufacture the particular product. Despite being extremely specific and impeachable, these techniques destroy the structure of food, are labor expensive, difficult, and can only be used on a laboratory scale. As a result, the need of the hour is to uncover alternative instrumentation techniques that can help overcome the majority of the restrictions that traditional methods have to offer. Spectroscopy is a developing method for certifying the authenticity of food products that is quick, low-cost, and non-destructive. Table 3 depicts a comprehensive review of various research studies and their findings in relation to spectroscopic techniques utilized for authentication of milk and milk products.

**TABLE 3:** Spectroscopic techniques for authentication/adulteration of milk and milk products.

Milk and milk products	Issue related to authentication	Spectroscopic technique employed	Data analysis/Chemometric analysis	Experiment conditions	Outcomes	References
Goat milk	Adulteration with cow milk	Near-infrared (NIR) spectroscopy	PLS algorithm	7 lots with 18 samples of goat milk and bovine milk separately Spectra was documented at a resolution of 8 cm^–1^and integration of 32 scans	Successfully detected the adulteration of goat milk with cow milk even at a minimum concentration of 1.0154 per 100 grams	(158)
Camel milk	Adulteration with goat milk	NIR spectroscopy (wavelength ranging from 700 to 2500 nm, resolution at 2 cm^–1^)	Multivariate analysis	03 samples of camel milk adulterated with goat milk at different concentrations	Could detect the goat milk up to 0.5% whereas the limit of quantification of adulteration was 2.0% having *R*^2^ value 94%.	(159)
Cow Milk	Adulteration	NIR spectroscopy (64 scans at 8 cm^–1^ resolution)	Standard variance spectrum of precision tests	Approximately 800 milk samples from different regions of China. Out of 800, 287 samples were of raw cow milk and remaining 526 of adulterated milk with thickeners and pseudo proteins (melamine, ammonia and urea)	Water signal in NIR spectra of milk was found to be crucial component determining contaminated milk discrimination.	(160)
Milk	Diagnosis of mastitis and the disease-causing microorganisms	NIR spectroscopy (spectra ranged from 400 to 2500 nm; 2 nm interval)	PLSR	200 numbers of foremilk samples taken from morning as well as afternoon milking	Could be employed for *in vivo* tracking of health of animal by somatic cell count, recognition of pathogens	(161)
Liquid milk	Melamine adulteration	NIR spectroscopy	One-class partial least squares	No. of samples 102 NIR spectrum- 32 scans; 1557 points	The results depicted that melamine adulteration could be depicted up to an accuracy of 89%, sensitivity 90% with 88% specificity	(151)
Cheese	Presence of goat, cow, ewe milk	NIR spectroscopy	PCA, MPLS	No. of samples 112 NIR spectrum scan 1100 to 2000 nm for every 2 nm 699 data points per sample	Was able to identify the fatty acid composition, thereby predicting the variability in the type of milk used for its production	(150, 162)
Cow milk market samples	Adulterants including hydrogen peroxide, urea, whey, synthetic milk	Attenuated total reflectance Fourier Transform Infrared spectroscopy (FTIR) (wavelength between 4000 to 500 cm^–1^)	SIMCA and PLSR	No. of samples 370 Spectra range 4000 to 700 cm^–1^; Resolution 4 cm^–1^; No of scans 128	With the help of Soft Independent Modeling of Class Analogy (SIMCA) the limits up to which adulterants like hydrogen peroxide (>0.019 g/L), urea (>0.78 g/L), whey (>7.5 g/L), and synthetic milk (>0.1 g/L) are added could be identified.	(163)
Liquid milk	Sucrose adulteration	Attenuated total reflectance Fourier Transform Infrared spectroscopy	Multivariate analysis (PCA and SIMCA)	No of scans 32/sample; Resolution 4 cm^–1^ Spectral range 4000–400 cm^–1^	Coefficient of determination value obtained was 0.996 Detection limit (DL) - 0.5%	(164)
Liquid Milk	Authentication of cow feeding and geographical origin	NIR spectroscopy	Cluster Analysis and PLS discriminate	No. of samples 486 Scan ranged from 400 to 2498 nm with 2 nm interval No. of scans 32	Could easily distinguish the milk obtained from pasture fed and preserved forage fed animals which was reflected by low error rate of 5.4% even for the diet having lower proportion of pasture (30%) whereas error was stable (2.5%) when pasture proportion was more than 70%	(152)
Ghee	To detect lard in pure ghee	Attenuated total reflectance Fourier Transform Infrared spectroscopy	Chemometrics	Wavelength between 4000 to 500 cm^–1^	Percentage accuracy was more than 99% Could detect lard even at 3% level also	(165)
Milk powder	Exogenous proteins adulteration	LIBS	Convolutional neural network (CNN)	−	CNN helped in achieving a high accuracy rate of 97.7% and prediction rate of 97.8%	(152)
Butter	Margarine adulteration	LIBS	PLS and PCA	12 and 5 samples of butter and margarine Laser operated in Q switched mode with rate of repetition, gate delay and an integration time of 8Hz, 650 ns and 1.05 ms, respectively.	Exhibited very little error rate of prediction to be 3.37 while the error rate for calibration was 2.02 DL – 3.9% Quantitation limit (QL)- 11.8%	(166)
Infant milk	Melamine	LIBS	PCA, univariate and NN	10,04, 04, and 02 pure semi-skimmed milk, branded cow milk, goat and sheep milk sample, respectively Laser pulse energy 100 mJ Integration time 20 ms	Generated finer results in comparison to the traditional techniques *R*^2^ values for univariate and NN model were quoted as 0.982 and 0.999, respectively	(167)
Butter	Margarine adulteration	Raman spectroscopy	PLS, PCA, principal component regression (PCR), artificial neural networks (ANNs)	No. of samples 01-homemade, 07- commercial, 02- regular margarine and 04- light margarine Spectral range 200–2000 cm^–1^ Resolution 2 cm^–1^; acquisition time10 s; No of measurements per sample- 02; Laser power 100 mW	R^2^ values for PLS, PCR and ANN were 0.987, 0.968, and 0.978, respectively	(168)
Milk powder	Melamine adulteration	Hyperspectral near-infrared imaging	Spectral angle measure (SAM), spectral correlation measure (SCM), and Euclidian distance measure (EDM)	No. of samples 36 (replicated); Spectra range 950–1700 nm	Less than 1% adulteration could be detected in milk powders	(169, 170)
Cheese	Starch adulteration	Hyperspectral near-infrared imaging	PLSR	Spectra range 200–1000 nm	Reported *R*^2^ value was 0.9915	(171)
Buffalo milk	Cow milk adulteration	Synchronous fluorescence (SF) spectroscopy	PCA, PLS	−	DL-6%	(172)

## Future recommendations and conclusion

This paper has reviewed the latest literature about different spectroscopic techniques used to resolve issues related to authenticity and adulteration of animal-based products. There has been an upsurge in the array of applications combining advanced spectroscopic techniques with modern multivariate data analytics, according to the published literature in the field. The research investigations revealed that these spectroscopic techniques are convenient to use, non-destructive, have no special requirements for sample preparation, and provide rapid, precise, and reproducible results. However, data obtained from the spectroscopic is then subjected to multivariate analysis, i.e., different statistical and chemometric tools for better classification of adulterated samples as well as the authenticity of original food products. To resolve this limitation, several methodologies referred to as chemometrics or data analytics are critical for the future successful development and implementation of new prediction models. Despite being repeatable and seldom impacted by variations in sensitivity over time, vibrational spectroscopy, NMR, and fluorescence spectroscopy. In fact, there are no particular prerequisites for sample preparation, which assure long-term stability and online or in-line application throughout the production process. This is especially the case for “homogeneous” liquid samples, whereas substantial sample preparation or repeated point measurements may be recommended for solid heterogeneous commodities like meat, fish, and dairy. According to the characteristics of the food product, choosing the right acquisition mode is also essential for obtaining accurate spectroscopic outcomes. This includes selecting the appropriate technique viz., NIR, IR, NMR, or fluorescence spectroscopy, sample presentation viz., transmission, absorbance, reflectance, excitation or emission, etc., type of sample holder viz., cuvette, probe, attenuated reflectance holder and working system. The spatial variability of components in heterogeneous goods can be differentiated using site-to-site spectroscopic fingerprint specificity, which makes HSI technologies a legitimate substitute for point spectral scanning. With regard to animal-based products in particular, HSI technologies using NIR radiation have been extensively utilized for food quality assessment and authentication. However, the majority of the documented works are laboratory-scale expediency, and there are few studies demonstrating the model’s reliability at the level of processing plants. High investment costs and challenging amortization for technology purchases are barriers to the widespread use of these spectroscopic technologies in the commercial context of the animal food business, particularly in small and medium farms where basic technologies are frequently unavailable. Another limitation is the need for calibration procedures, management, and interpretation of the data which also depends on the skill and knowledge of specialized personnel. The sample preparation for various techniques like HIS, and NIR serves as a rate-limiting step while analyzing a larger sample population for the characterization, as well as generation of enormous data for each individual measure and the comparatively lengthy processing durations for this data.

However, there are still several obstacles to overcome in terms of widespread adoption and deployment of these technologies in both academic and commercial facilities. This necessarily requires the creation of the next generation of scientists capable of working in both academia and industry contexts, and skilled in dealing with all aspects of food authentication using quasi techniques. When it comes to food verification, spectroscopic techniques have evident advantages over-focused approaches; nonetheless, their widespread implementation outside of laboratories remains a challenge. To address these difficulties, researchers will link new spectroscopic technologies with the demands of food fraud risk management systems, paving the way for their use in food assurance. For the entire spectroscopic domain, and particularly for those that are more focused on food verification and authentication, it is required to fabricate simplified, compact, and portable instruments. Therefore, it is apparent that spectroscopic techniques offer significant advantages over focused approaches when dealing with a food authentication issue; nonetheless, their widespread implementation outside of laboratories still poses difficulties. By overcoming these obstacles, developing spectroscopic techniques will be matched with the requirements of food fraud risk management systems, opening the door for their application to the assurance of food integrity.

## Author contributions

VC and PK contributed to concept and writing original draft. AD, CM, and CS contributed to writing original draft. RP contributed to resources and writing – original draft, review and editing. All authors contributed to the article and approved the submitted version.
